# High-Resolution Molecular Epidemiology and Evolutionary History of HIV-1 Subtypes in Albania

**DOI:** 10.1371/journal.pone.0001390

**Published:** 2008-01-02

**Authors:** Marco Salemi, Tulio de Oliveira, Massimo Ciccozzi, Giovanni Rezza, Maureen M. Goodenow

**Affiliations:** 1 Department of Pathology, Immunology, and Laboratory Medicine, University of Florida, Gainesville, Florida, United States of America; 2 Medical Research Council (MRC) Bioinformatics Unit, South African National Bioinformatics Institute, University of Western Cape, Cape Town, South Africa; 3 Istituto Superiore di Sanita', Roma, Italy; University of California at Berkeley, United States of America

## Abstract

**Background:**

HIV-1 epidemic in Western Europe is largely due to subtype B. Little is known about the HIV-1 in Eastern Europe, but a few studies have shown that non-B subtypes are quite common. In Albania, where a recent study estimated a ten-fold increase of AIDS incidence during the last six years, subtype A and B account for 90% of the know infections.

**Methodology/Principal Findings:**

We investigated the demographic history of HIV-1 subtype A and B in Albania by using a statistical framework based on coalescent theory and phylogeography. High-resolution phylogenetic and molecular clock analysis showed a limited introduction to the Balkan country of subtype A during the late 1980s followed by an epidemic outburst in the early 1990s. In contrast, subtype B was apparently introduced multiple times between the mid-1970s and mid-1980s. Both subtypes are growing exponentially, although the HIV-1A epidemic displays a faster growth rate, and a significantly higher basic reproductive number R_0_. HIV-1A gene flow occurs primarily from the capital Tirane, in the center of the country, to the periphery, while HIV-1B flow is characterized by a balanced exchange between center and periphery. Finally, we calculated that the actual number of infections in Albania is at least two orders of magnitude higher than previously thought.

**Conclusions/Significance:**

Our analysis demonstrates the power of recently developed computational tools to investigate molecular epidemiology of pathogens, and emphasize the complex factors involved in the establishment of HIV-1 epidemics. We suggest that a significant correlation exists between HIV-1 exponential spread and the socio-political changes occurred during the Balkan wars. The fast growth of a relatively new non-B epidemic in the Balkans may have significant consequences for the evolution of HIV-1 epidemiology in neighboring countries in Eastern and Western Europe.

## Introduction

Human immunodeficiency virus type 1 (HIV-1) infection continues to spread rapidly throughout the world. According to the UNAIDS/WHO (June 2006) report, an estimated 39.5 million people are living with the virus worldwide. HIV-1 is characterized by high genetic variability, rapid evolution, and diversification [1], [2]. Indeed, recombination coupled with the elevated error rate of the reverse transcriptase, and the rapid turnover of HIV-1 in infected individuals, are at the origin of the high genetic variability of the virus [3].

The majority of HIV-1 strains cluster within a large group called M (for Main), which is responsible for the pandemic [3]. Group M includes ten phylogenetically distinct subtypes, two sub-subtypes, and several inter-subtype HIV-1 recombinants, known as circulating recombinant forms (CRFs) [4]–[6]. Most subtypes, as well as CRFs, are present in Africa, reflecting the African origin of the epidemic during the 1930s [7]–[9]. HIV-1 subtype C is the most prevalent worldwide accounting for more than half of all infections, whereas HIV-1B is the subtype responsible for most of the infections in Western Europe, United States, and Australia [10]. Little is known about the HIV-1 epidemic in Eastern Europe, but a few studies have shown that non-B subtypes are quite common. For example, HIV-1A is highly prevalent in Russia, Belarus, and Ukraine [11]–[13], as well as former Yugoslavia [14]. In Albania, a recent report has shown the presence of both HIV-1A and B subtypes, which account for about 90% of the known infections [15]. Although the registered HIV-1 cases in Albania are still low the social, political, and economical instability of the country has its own risk determinants with regard to sexually transmitted diseases in general and for HIV/AIDS in particular. A detailed characterization of the demographic parameters shaping the Albanian epidemic, including the growth rate in the number of effective infections, the time of introduction of a lineage in a population, and the viral gene flow (migration) to/from neighboring countries, has important implication for the evolution of the HIV-1 epidemic in the European continent as a whole.

Phylogenies reconstructed from randomly sampled viral gene sequences contain valuable and unique information about population-level processes such as change in population size and growth rate, and can be used to understand the course of a viral epidemic over time [16], [17]. Indeed, coalescent theory has successfully been employed to infer the history of a pathogen population and investigate the evolutionary dynamics of different HIV-1 subtypes within a specific geographic region [16]–[20]. To this end, it has been demonstrated that the *pol* gene contains sufficient evolutionary information to reconstruct demographic histories in spite of the potential bias introduced by the emergence of drug resistance mutations [20], [21]. Moreover, the Albanian data set included only therapy-naïve subjects and no major mutations associated with drug resistance were identified in such a cohort [15]. The coalescent framework also assumes neutral evolution and several studies have shown that the *pol* gene is under both positive and negative selection [22]–[25]. However, even when an HIV-1 gene evolving within a host is under strong selection, the genealogies generated at the epidemiological (inter-individual) level appear to be neutral and do not significantly violate the assumptions of the coalescence [26]. In fact, even when different HIV-1 genes undergo significantly different selection pressure remarkably similar and reliable demographic estimates are still obtained from coalescent analysis [18].

In the present study we used high-resolution phylogenetic analysis and the coalescent framework, as well as a phylogeographic approach [27], to reconstruct the history and evolution of HIV-1A and HIV-1B epidemics in Albania. Our data not only provide important insights about the extent of the epidemic in this country, but also indicate how, in a time of intense socio-political change and migration trends, the emergent epidemics in the Balkans may have profound implications for the future of HIV-1 epidemiology in Europe and worldwide.

## Results

### Origin of HIV-1A and HIV-1B Albanian epidemics

**Figure 1:**
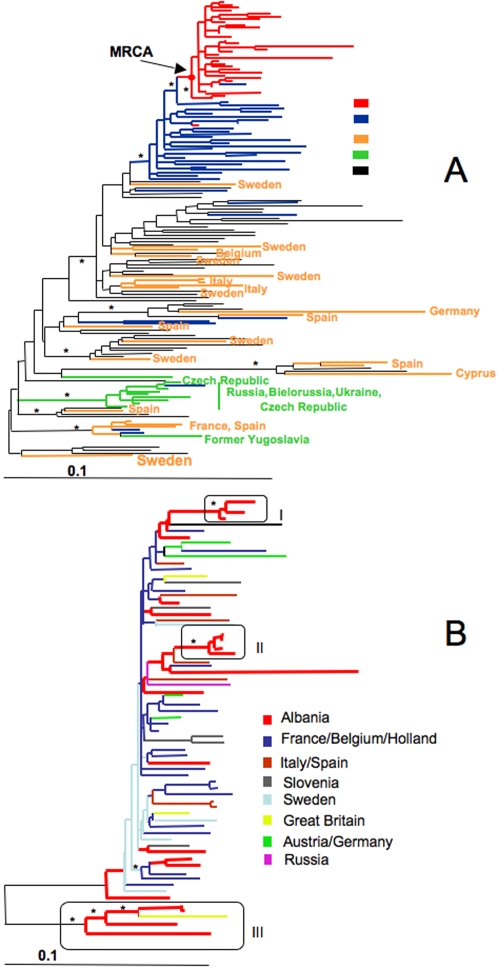
Maximum likelihood phylogenetic analysis of HIV-1A pol sequences. Branch lengths were estimated with the best fitting nucleotide substitution model according to a hierarchical likelihood ratio test [29], and were drawn in scale with the bar at the bottom indicating 0.1 nucleotide substitutions per site. One * along a branch represents significant statistical support for the clade subtending that branch (p<0.001 in the zero-branch-length test and bootstrap support >75%). The color of each tip branch represents the country (or geographic region) of origin of sequence corresponding to that branch, according to the legend in the figure. A. Phylogenetic tree of 31 HIV-1A strains from Bulgaria and 122 subtype A reference sequences downloaded from the Los Alamos HIV database. The tree was rooted using two HIV-1B strains as outgroup. The arrow indicates the most recent common ancestor (MRCA) of the Albanian monophyletic clade. B. Phylogenetic tree of 21 HIV-1B strains from Bulgaria and 46 subtype B reference sequences downloaded from the Los Alamos HIV database. The tree was rooted using two HIV-1A strains as outgroup. Solid boxes highlight statistically supported clades of Albanian sequences.

To investigate the origin of HIV-1 subtype A and B in Albania we compiled different data sets of Albanian and reference sequences from different geographic origin (supplemental Table S1). Phylogenetic analysis of HIV-1A and HIV-1B Albania+reference sequences alignments pointed to different origins for the two subtypes in the Balkan country. HIV-1A ML inferred genealogy (Figure 1A) clustered all Albanian strains but one within a monophyletic clade highly supported both by boostrapping (>70%) and zero branch length test (*p*<0.001). The Albanian clade emerged as a subcluster of a monophyletic group joining sequences from Greece and also including one Albanian strain (the ML genealogy displaying full sequence names is given in supplemental Figure S1A). The finding that 99% of the HIV-1A Albanian sequences could be traced back to a unique most recent common ancestor (MRCA) suggested a single major introduction of HIV-1A from Greece followed by local epidemic spread. The presence of one Greek sequence within the Albanian clade and viceversa (see Figure 1A) could also indicate a limited ongoing viral gene flow between the two neighboring countries. HIV-1B ML inferred genealogy (Figure 1B) showed Albanian sequences highly scattered among strains collected from several other European countries. Three well-supported Albanian clades (bootstrap >75% and the zero branch length test *p*<0.001) are visible in the tree. Each clade belonged to a separate phylogenetic lineage supporting a scenario of multiple independent introductions to Albania of subtype B from different geographic areas (the ML genealogy displaying full sequence names is given in supplemental Figure S1B). The existence of several independent Albanian clades was also an indication of separate transmission networks, originating from different introductions of the virus at different times, within the country.

### Likelihood mapping analysis

**Figure 2:**
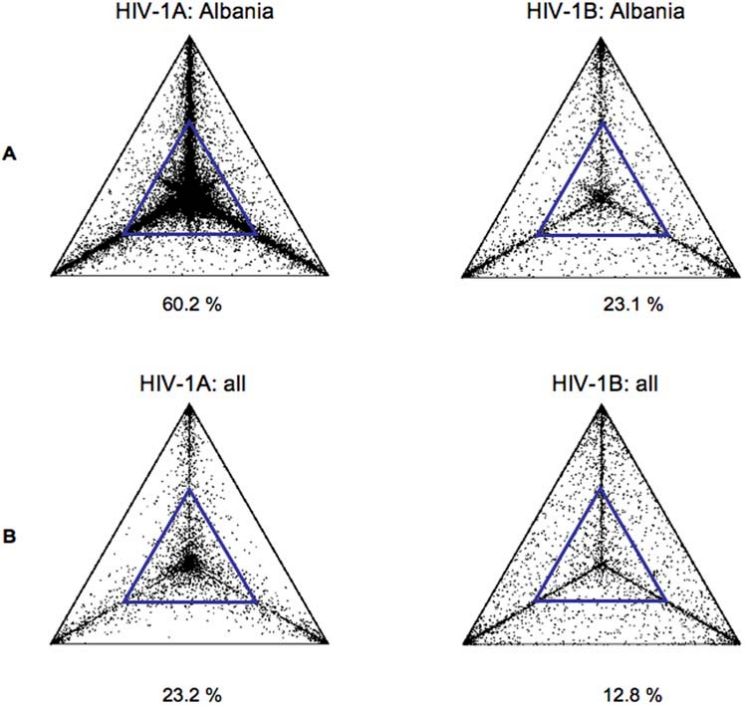
Likelihood mapping of HIV-1A and B pol sequences. Each dot represents the likelihoods of the three possible unrooted trees for a set of four sequences (quartets) selected randomly from the data set (see Materials and Methods): dots close to the corners or the sides represent, respectively, tree-like, or network-like phylogenetic signal in the data. The central area of the likelihood map, highlighted by a smaller blue triangle inside the map, represents star-like signal. The percentage of dots in the central area is given at the basis of each map. A. Likelihood mapping of 10,000 random quartets of HIV-1A (left) and HIV-1B (right) Albanian sequences. B. Likelihood mapping of 10,000 random quartets of HIV-1A (left) and HIV-1B (right) Albanian+reference sequences downloaded from the Los Alamos HIV databases.

The phylogenetic structure of the Albanian epidemics was also investigated by likelihood mapping analysis. The evaluation of 10,000 random quartets with the likelihood-mapping method showed a significant difference between HIV-1A and HIV-1B epidemic. More than 60% of the randomly chosen quartets from the HIV-1A alignment of Albanian strains were distributed in the center of the likelihood map, indicating a strong signal for star-like phylogeny (Figure 2A, left panel). In contrast, only 23.1% of quartets from the HIV-1B Albanian alignment fell in the center of the map with most of the quartets equally distributed in the three corners, which represent tree-like signal (Figure 2A, right panel). On the other hand, HIV-1A and HIV-1B maps in the rest of Europe (after excluding the Albanian sequences) exhibited very similar structure, although subtype A still showed a larger star-like signal with respect to subtype B (Figure 2B). The detailed distribution of dots in each region of the likelihood mappings is given in supplemental Figure S2.

### Demographic history of HIV-1 Albanian A and B subtypes

**Figure 3:**
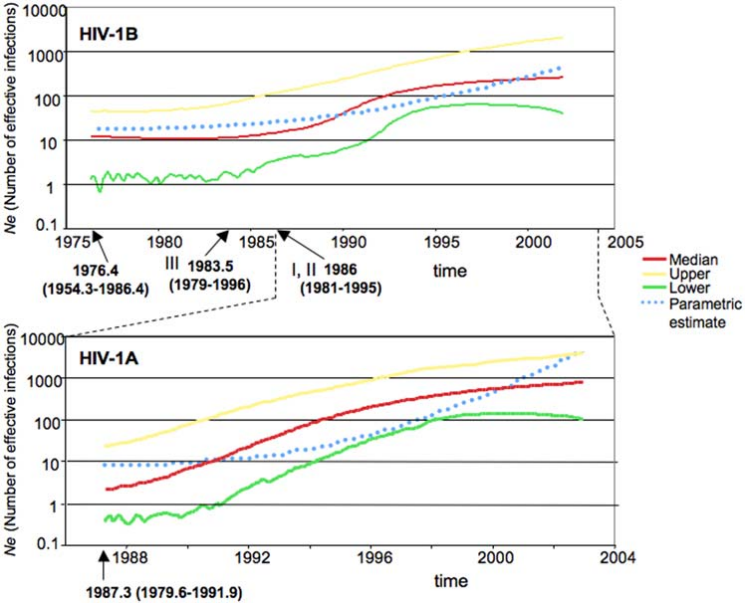
Bayesian skyline plots of HIV-1 subtypes in Albania. Non-parametric curves of HIV-1 effective population size (effective number of infections, Ne) over time in Albania were estimated from pol gene sequences employing a Bayesian framework. Genetic distances were transformed into a timescale of years taking into account the different sampling times of the viral strains and by enforcing a relaxed molecular clock model (see Materials and Methods). Solid lines indicate median, and 95% upper and lower high posterior density (HPD) estimates of Ne, according to the color legend to the right; dotted blue lines are parametric estimate of Ne(t) according to the exponential demographic model. Broken lines between the top and bottom panels highlight the time window common to the two epidemics. Top panel. HIV-1 subtype B. Each arrow indicates the estimated origin of the corresponding Albanian clade (indicated by the roman numeral) in the tree in Figure 1B. The first arrow from the left indicates the time of the MRCA. 95% HPD intervals are given in parenthesis below each estimate. Bottom panel. HIV-1 subtype A. The arrow indicates the estimated origin of the MRCA of the Albanian clade shown by the tree in Figure 1A. 95% HPD intervals are given in parenthesis below each estimate.

HIV-1A and HIV-1B Albanian sequences were analyzed separately to infer the demographic history of the two subtypes within the Balkan country. We calculated approximate marginal likelihoods of six different demographic models (supplemental Table S2): four parametric models (constant population size with strict or relaxed molecular clock, exponential growth with strict or relaxed molecular clock), and two non-parametric models (Bayesian skyline plot, BSP, with strict or relaxed molecular clock). For both data sets, the Bayes Factor (BF) favored, in general, models enforcing a relaxed molecular clock over strict clock models (Table 1). Models assuming exponential population growth performed always better than models assuming constant population size. In contrast, the BF was not significant when exponential models were compared with BSPs (Table 1) suggesting that both the parametric and the non-parametric models fit the data equally well. The BSP of HIV-1A dated the origin of the MRCA of the Albanian epidemic in the late 1980s (Figure 3, bottom panel) with an initial median estimate of 4 effective infections (95% high posterior density interval, 95% HPD, 0.4 to 22). The final median estimate in the year 2003 (the latest sampling date available) resulted of about 757 effective infections (95%HPD = 101–6,000). The reconstruction of HIV-1B demographic history estimated the mid-1970s as the origin of the MRCA (Figure 3, top panel). However, It is important to keep in mind that because HIV-1B Albanian strains were scattered among other European sequences, such a date indicated rather the origin of the MRCA of HIV-1 in Europe than in Albania where, in fact, multiple independent introductions over time were postulated. The origin of the Albanian transmission clades within the HIV-1B tree (Figure 2) was traced back between 1983 and 1986 (Figure 3, top panel). The median estimate of *Ne* in the year 2002 (the latest sampling date available) resulted of about 243 effective infections (95%HPD = 36–1,919), about one third of the estimate for HIV-1A and in substantial agreement with epidemiological data showing HIV-1A as the prevalent infection [15]. Moreover, comparison of HIV-1A and HIV-1B demographic histories showed that subtype B epidemic expanded initially at a much slower than the HIV-1A epidemic. Both parametric and non-parametric subtype B demographic curves looked approximately linear during the first decade of the epidemic, and experienced a sharper increase only within the next fifteen years during the same time-frame of the introduction and exponential growth of subtype A epidemic (Figure 3). In fact, by 1996, nine years after the first introduction in Albania of HIV-1A, the effective number of subtype A infections was already larger than the one due to HIV-1B.

**Table 1:** Bayes Factors between different demographic models of HIV-1A and B in Albania.

subtype	Model comparison^1^	2·BF^2^	Evidence against H_0_ 3
**HIV-1A**	Const Strict (H_0_) vs Relaxed (H_1_) clock	46.6 (0.104)	very strong
	Expo Strict (H_0_) vs Relaxed (H_1_) clock	63.5 (0.96)	very strong
	BSP Strict (H_0_) vs Relaxed (H_1_) clock	48.44 (0.92)	very strong
	Const (H_0_) vs Expo (H_1_) Relaxed clock	8.58 (0.104)	strong
	BSP (H_0_) vs Expo (H_1_) Relaxed clock	2.22 (0.97)	weak
**HIV-1B**	Const Strict (H_0_) vs Relaxed (H_1_) clock	20 (0.116)	very strong
	Expo Strict (H_0_) vs Relaxed (H_1_) clock	20 (0.92)	very strong
	BSP Strict (H_0_) vs Relaxed (H_1_) clock	20 (0.1)	very strong
	Const (H_0_) vs Expo (H_1_) Relaxed clock	8.58 (0.104)	strong
	BSP (H_0_) vs Expo (H_1_) Relaxed clock	2.22 (0.97)	weak

1. Cost = costant population size; Expo = exponential population growth; BSP = Bayesian Skyline Plot; Strict = strict molecular clock; Relaxed = relaxed molecular clock.

2. BF = Bayes Factor is the difference (in log_e_ space) of the marginal likelihood of null (H_0_) and alternative (H_1_) model. BFs were estimated by comparing the approximate marginal likelihoods of different models given in Table S2. The standard error of the estimate is given in parenthesis.

3. Evidence against H_0_ is assessed in the following way: 2>2 BF>6 indicates positive evidence against the null model; 6>2·BF>10 indicates strong evidence against the null model; 2·BF>10 indicates very strong evidence against the null model.

### Grow rate estimates of HIV-1 subtype A and B Albanian epidemics

**Figure 4:**
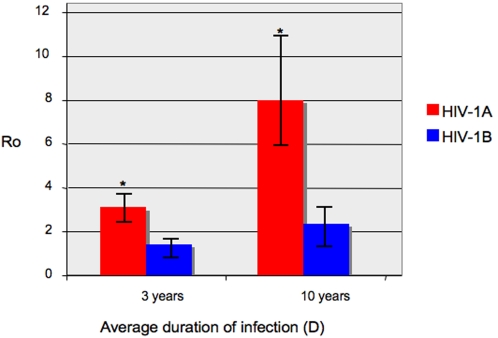
Estimates of HIV-1A and HIV-1B basic reproductive number (R0) in Albania. R0 estimates for different average duration of infection (D) were inferred from the Bayesian estimates of population growth rate (see materials and Methods). A Black bar indicates the 95% high posterior density intervals of the estimate. An * on top of the bar indicate a statistically significant difference (paired t-test, p<0.01) between R0 estimates for two different subtypes.

Under the exponential model, the effective number of infections *Ne* grows exponentially at rate *r*. It has to be noted that *Ne* represents the number of infections actually contributing to new infections, rather than the total number of infected individuals. Such observation is reflected by the estimates of the growth rates that resulted 0.69 years^−1^ (95%HPD = 0.47–0.905) for HIV-1A, but only 0.136 years^−1^ (95%HPD = 0.064–0.205) for HIV-1B. Basic reproductive numbers (R_0_) of HIV-1A and HIV-1B in Albania, obtained from the parametric estimates of the growth rate *r*, was also significantly different (*p*<0.01, paired t-test) for the two subtypes (Figure 4). Assuming the virus is transmitted at the same rate during the total length of infection, a short term progressor would transmit the infection 1 to 2 times if infected by HIV-1B, but 2 to 4 times if infected with HIV-1A. An HIV-1A long term progressor would transmit the infection 6 to 8 times compared to the 1 to 3 times of an HIV-1B long term progressor.

### Phylogeography of HIV-1 Albanian subtypes

**Figure 5:**
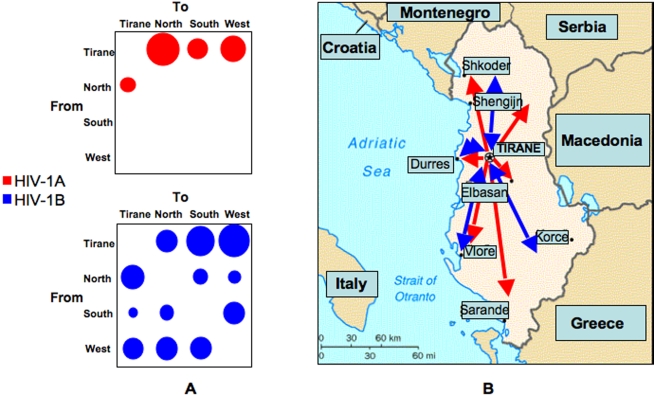
Phylogeographic mapping of HIV-1A and B epidemic in Albania. A. The bubblegrams show the frequency of gene flow (migrations) in Albania to/from different geographic areas and the country capital Tirane. The surface of each circle is proportional to the percentage of observed migrations in the ML genealogy (supplemental Figure S3). Migrations were inferred with a modified version of the Slatkin and Maddison algorithm [27], [41] for the HIV-1A (top panel) and HIV-1B (bottom panel) subtype from the maximum likelihood inferred genealogies given in Figure 1 and 2, respectively. B. Detailed mapping of HIV-1A (indicated by the red arrows) and HIV-1B (indicated by the blue arrows) gene flow among different Albanian geographic areas.

The gene flow (migration) of HIV-1 subtype A and B among different geographic areas and major cities in Albania was investigated with a modified version of the Slatkin and Maddison method [27]. After superimposing the city of origin of the Albanian sequences on the tip branches of the ML genealogy, we inferred the city of origin of each ancestral node (i.e. ancestral sequence) in the tree using the maximum parsimony algorithm (supplemental Figure S3). The general migration trend between Tirane, the capital roughly at the center of the country, and the major cities at North, South, and West of Tirane was then estimated as observed migration in the genealogy (Figure 5A). The null hypothesis of panmixia (i.e. no population subdivision or complete intermixing of sequences from different geographic areas) was rejected by the randomization test [27] for subtype A (*p*<0.001), but not for subtype B (*p* >0.05). Subtype A gene flow appeared highly asymmetrical with the virual infections mainly expanding from the center to the periphery (Figure 5A top panel). In contrast, HIV-1B flow appeared symmetrical with no preferred migration route (Figure 5A bottom panel). Tirane appeared to be the epicenter of HIV-1A epidemic, exporting the infections toward most of the major cities in the country (Figure 5B).

## Discussion

The application of high-resolution phylogenetic methods and the coalescence as tools to study the evolutionary dynamics of pathogens both within and among hosts has been undergoing a dramatic development within the recent years [26]. The present study is an example of the detailed epidemiological and evolutionary information that can successfully be extracted from sampled gene sequence data. By employing both a coalescent framework and a phylogeographic approach we were able to investigate the origin and demographic history of HIV-1A and HIV-1B epidemics in Albania to a significant depth. A recent survey of different subtypes circulating in the Balkan country has shown that HIV-1A and B were responsible for about 90% of the known infections [15]. The study pointed out that subtype A prevalence increased over time and became predominant even if, surprisingly, in neighboring countries hosting a large number of Albanian immigrants, such as Italy and Greece, subtype B is still the predominant epidemic. Our data demonstrated that while HIV-1B epidemic in Albania has been characterized by multiple introductions throughout the 1980s and a relatively slow growth, HIV-1A has been spreading, right after its limited introduction in the late 1980s, at an explosive rate. Demographic curves and likelihood mappings were both consistent with a star-like outburst of subtype A during a relatively short time interval. In contrast, subtype B epidemic appeared more structured and characterized by discrete transmission clusters evolving as independent phylogenetic lineages over a longer period of time. The reconstruction of HIV-1 demographic history in the Balkan country coupled with phylogeographic analysis pointed out how complex socio-political and economic changes can dramatically influence the epidemiology of a newly introduced pathogen in a population. The Wars in the Balkans resulted in significant loss of human life, infrastructure damage and the severe disruption of social and health services, especially those supporting women and children. In 1995 over half of Albanian population were refugees or displaced. The war created conditions favorable to HIV/AIDS transmission. The breakdown in the health and public services together with increased risk behaviors including substance abuse, commercial sex work, and sexual behavior in young people are enabling factors for increased HIV/AIDS transmission. An inadequate public health and public awareness programs could have significantly contributed to the spread of HIV-1 among risk groups. The early 1990s were the years when, according to our estimates, both subtype A and B began to spread fast with a considerable gene flow from the capital Tirane, the most developed city in the center of the country, to the periphery. However, the estimated growth rate of subtype A resulted five times larger than subtype B. Such finding does not imply anything about increased pathogenicity of subtype A that, although it cannot be excluded, has not been substantiated so far by any other data. The expansion of both epidemics can be traced back to the dramatic socio-political changes occurred in Albania during the early 1990s. Their subsequent different growth, with HIV-1A becoming quickly the predominant infection, may be due to their introduction in different susceptible populations living in urban or rural areas. A similar explanation has been proposed for the faster growth rate of HIV-1 subtype C with respect to subtype B in Southern Brazil [20]. It is important to notice that although R_0_ estimates for subtype A and B are in agreement with this scenario, they should be taken with caution, since they rely on the assumption that the virus is transmitted at the same rate during the total length of infection. Such an assumption seems to be reasonable for pathogens like HCV [16], but it is less clear whether it could be valid for HIV-1 as well.

The only epidemiological survey in Albania conducted so far at the national level, of which we have used the molecular data analyzed in the present work, collected 72 plasma samples representing about 50% of the country's known individuals with HIV/AIDS [15]. The study estimated an increase in the incidence of AIDS from 0.3 cases/million population in 1996 to 3.3 cases/million population in 2002. Although a ten-fold increase is in agreement with our overall finding, the parametric estimates of HIV-1A and B demographic histories suggested a much larger number of effective infections. Moreover, the number of effective infections (i.e. those one contributing to onward transmission) is always significantly lower than the total number of infected individuals. As an example, the number of effective infections in 1995 U.S.A reached 5,000, while the prevalence was actually 200,000 [17]. If a similar relationship held for the Albanian epidemics, our estimates would indicate the presence of about 30,000 people infected by subtype A and about 10,000 by subtype B in a country of about 3.6 millions people. Even if these numbers may be overestimates due to a bias in the coalescent models used, it has to be considered that to date national surveillance mechanisms and reporting systems in Albania are still underdeveloped resulting in a lack of adequate epidemiological information and behavioral data. Our analysis suggested that the extent of HIV-1 epidemic in Albania is much larger than previously thought with HIV-1A infections growing at an alarming rate. Through phylogeographic analysis we have shown that HIV-1A gene flow has been occurring form the capital Tirane to the cities of Vlore and Durres, the two major Albanian ports on the Adriatic sea close to Southern Italy, Shkoder, Northern city close to Montenegro, and Sarande, Southern city close to the Greek border. Such a trend, coupled with the significant Albanian emigration during the last recent years due to high rate of unemployment has the potential to introduce and spread a new wave of HIV-1A epidemic into Southern Europe significantly affecting, on the long run, HIV-1 epidemiology in Europe as well as other Western countries.

In conclusion, we showed how the coalescent and phylogeographic framework could be employed to investigate in detail the demographic history and the complex factors underlying the spread of pathogens in a population. Our findings highlight the dramatic influence that social, political, and economical factors can have in determining the outcome of a pathogen newly introduced in a susceptible population, and call for considerable improvements in prevention campaigns and monitoring of the HIV-1 infection in the Balkans.

## Materials and Methods

### Data sets

Two main data sets of previously published HIV-1 *pol* sequences (protease+RT) from Albania were compiled including, respectively, 31 HIV-1A strains collected between 1997 and 2003, and 21 HIV-1B strains collected between 1995 and 2002 [15]. Two additional data sets were obtained using subtype A and B reference sequences downloaded from the Los Alamos HIV databases (http://hiv-web.lanl.gov/). Full details on the alignments compiled for the present study are given in supplemental Table S1. To assemble data sets suitable for demographic analysis (see below), reference strains from epidemiologically unlinked individuals were chosen according to the following criteria: 1. known country were the infection was acquired; 2. known year of sampling, and 3. sequences from therapy naïve subjects and/or without known mutations associated with drug-resistance. Sequences were aligned with the Clustal algorithm [28] followed by manual editing. Positions containing gaps were removed from the final alignment. Alignments are available from the authors upon request.

### Phylogenetic analysis

For each data set, the best fitting nucleotide substitution model was tested with a hierarchical likelihood ratio test following the strategy described by Swofford and Sullivan (2003) [29], using a neighbor-joining (NJ) base-tree with LogDet corrected distances. Maximum likelihood (ML) trees were then inferred with the selected model and ML-estimated substitution parameters. Model and model parameters for each data set are given in supplemental Table S1. The heuristic search for the ML tree was performed using an NJ tree as starting tree and the TBR branch-swapping algorithm. NJ trees were also estimated using pair-wise distances inferred by ML with the best fitting nucleotide substitution model. Calculations were performed with PAUP* 4.0b10 written by David L. Swofford. Statistical support for internal branches in the NJ trees was obtained by bootstrapping (1000 replicates) and with the ML-based zero branch length test for the ML trees [29]. Trees were rooted by ML rooting by selecting the rooted tree with the best likelihood under the molecular clock constraint (taking into account the different sampling times of the *taxa*) [30], [31], or by outgroup rooting using two Albanian HIV-1A strains as outgroup for the HIV-1B data set, and two HIV-1B Albanian strains for the HIV-1A data sets. Both methods inferred the same root for each data set.

### Likelihood mapping

The phylogenetic signal in a data set of aligned DNA or amino acid sequences can be investigated with the likelihood mapping method by analyzing groups of four sequences, randomly chosen, called quartets [32]. For a quartet, just three unrooted tree topologies are possible. The likelihood of each topology is estimated with the maximum likelihood method and the three likelihoods are reported as a dot in an equilateral triangle (the likelihood map). Three main areas in the map can be distinguished as shown in supplemental Figure S2 [32]: the three corners representing fully resolved tree topologies, i.e. the presence of tree-like phylogenetic signal in the data; the center, which represents star-like phylogeny, and the three areas on the sides indicating network-like phylogeny, i.e. presence of recombination or conflicting phylogenetic signals. For N sequences 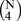 possible quartets exist and that the distribution (percentage) of dots within each area gives an idea about the mode of evolution in the data set under investigation. Extensive simulation studies have shown that >33% dots falling within the central area indicate substantial star-like signal, i.e. a star-like outburst of multiple phylogenetic lineages [32]. Likelihood mapping analyses were performed with the program TREE-PUZZLE [33] for each data set by analyzing 10,000 random quartets.

### Evolutionary rates of HIV-1A and HIV-1B subtypes

For HIV-1B, a previous estimate of the evolutionary rate (3.55 10^−3^ nucleotide substitutions/site year) from a data set of 106 sequences sampled between 1983 and 2000 was available [17]. For HIV-1A, the evolutionary rate was inferred from the data (1.4 10^−3^ nucleotide substitutions/site year, 95% high posterior density interval 0.28–2.6 10^−3^) since sequences were sampled at different time points and a molecular clock with non-contemporaneous tips could be calibrated [31], as done by Hue and coll. for HIV-1B [17]. To this end, the evolutionary rate was estimated by employing the Bayesian Markov Chains Monte Carlo (MCMC) clock method [34] implemented in the BEAST software package (http://evolve.zoo.ox.ac.uk/beast/). As a first approximation, we estimated the evolutionary rate using the full data set, including Albanian as well as reference sequences, with a strict Bayesian MCMC clock [34]. For all the subsequent calculations implementing various demographic models (see below) the subtype specific rate was employed as mean fixed rate for models enforcing a strict molecular clock, and as mean fixed rate with exponentially distributed evolutionary rates as prior for models enforcing a relaxed molecular clock [34].

The Bayesian calculation consisted of three independent 10,000,000 generations MCMC with sampling every 1000^th^ generation. The three independent runs were combined with LogCombiner version 1.4. Convergence of the MCMC was assessed by calculating the effective sampling size (ESS) of the runs [35]. All parameter estimates showed significant ESS (>250).

### Coalescent models

By using coalescent theory, we can infer the demographic history of a population from the genealogical relationships of sampled individuals [36], [37]. A genealogy reconstructed from randomly sampled HIV sequences contains information about population-level processes such as change in population size, and growth rate [38]. Given a viral phylogeny *P* and a vector φ representing the parameters of a demographic model *N*(*t*), it is possible to calculate the log of the conditional probability *ln*[φ|*P*]. Bayesian estimates of φ can be found by MCMC sampling procedure [38]. We considered six demographic models for the HIV-1A and HIV-1B epidemic in Albania: constant population size with strict or relaxed molecular clock, exponential growth with strict or relaxed molecular clock, and Bayesian skyline plot (BSP) with strict or relaxed molecular clock. Both parametric (constant or exponential model) and non-parametric (BSP) estimates of demographic history were performed with BEAST version 1.4.1 by running one MCMC for 100,000,000 generations with sampling every 10,000^th^ generation. Each aligned data set was partitioned in 1^st^+2^nd^ and 3^rd^ codon positions and the parameters of the nucleotide substitution (HKY+Γ+I) and demographic model were estimated independently for ach partition. All parameter estimates showed significant ESS (>300).

### Bayesian selection of coalescent models

While the constant and the exponential model are nested, the BSP is a non-parametric model that cannot be compared with the other two by comparing the mean log posterior probabilities. Model comparison in a Bayesian framework can be achieved, however, by calculating the Bayes Factor (BF), which is the ratio of the marginal likelihoods (marginal with respect to the prior) of the two models being compared [39]. We calculated approximate marginal likelihoods for each coalescent model via importance sampling using the harmonic mean of the sampled likelihoods (with the posterior as the importance distribution) [40]. The difference (in log_e_ space) of marginal likelihood between any two models is the log_e_ of the Bayes Factor, log_e_(BF). Evidence against the null model (i.e. the one with lower marginal likelihood) is assessed in the following way [39]: 2>2· log_e_(BF)>6 indicates positive evidence against the null model; 6>2·log_e_(BF)>10 indicates strong evidence against the null model; 2·log_e_(BF)>10 indicates very strong evidence against the null model. The calculations were performed with BEAST 1.5 alpha (http://code.google.com/p/beast-mcmc/) according to the instructions on the BEAST website (http://beast.bio.ed.ac.uk/Model_comparison).

### Grow rate of HIV-1 subtype A and B epidemic

Since the exponential model was the one with the highest marginal likelihood for both HIV-1A and B Albanian data sets (see supplemental Table S2), we could use the population growth rate, *r*, which is one of the two free parameters (the other is the number of effective infections *Ne*) of the model to infer the epidemiological quantity R_0_. R_0_ is the basic reproductive number (infectivity) of a pathogen, i.e. the average number of secondary infections caused by each primary infected individual. In a viral population exponentially growing at rate *r*, with *D* as the average duration of infectiousness, it can be shown that if the virus is transmitted at the same rate during the total length of infection, then R_0_ = *rD*+1[16]. The limitations of such an assumption for HIV-1 are discussed in the Discussion section.

### Phylogeographic analysis

The hypothesis of restricted gene flow among distinct HIV-1 populations within different geographic regions and cities in Albania was tested with a modified version of the Slatkin and Maddison test [27] that allows to count migration to/from different viral subpopulations [41], using the MacClade version 4 program (Sinauer Associates, Sunderland, MA), as described below. A one-character data matrix is obtained from the original data set by assigning to each *taxon* in the tree a one-letter code indicating its country (or geographic region) of origin. Then, the putative origin of each ancestral sequence (i.e. internal node) in the tree is inferred by finding the most parsimonious reconstruction (MPR) of the ancestral character (supplemental Figure S3). The final tree-length, i.e. the number of observed migrations in the genealogy, can easily be computed and compared to the tree-length distribution of 10,000 trees obtained by random joining-splitting. Observed genealogies significantly shorter than random trees indicate the presence of subdivided populations with restricted gene flow [27]. Specific migrations among different countries (character states) were traced with the *State changes and stasis* tool (MacClade software), which counts the number of changes in a tree for each pair-wise character state. When multiple MPRs were present (as in our data sets), the algorithm calculated the average migration count over all possible MPRs for each pair. The resulting pair-wise migration matrix was then normalized, and a randomization test with 10,000 matrices obtained from 10,000 random trees (by random joining-splitting of the original tree) was performed to assess the statistical significance of the observed migration counts.

## Supporting Information

Table S1 HIV-1 pol gene data sets assembled for the present study.
(0.06 MB DOC)

Table S2 Marginal Likelihoods for different epidemiological models of HIV-1A and B in Albania.
(0.04 MB DOC)

Figure S1 Maximum likelihood phylogenetic analysis of HIV-1 A and B pol sequences. The maximum likelihood trees display the full names of the HIV-1 sequences used in the analysis. Two digits at the beginning of the name indicate the sampling year, and the next two characters the country of origin according to the guidelines at the Los Alamos HIV databases. Albanian sequences are indicated as “Alb”. A. The tree is the same as the one given in Figure 1A for HIV-1A pol sequences. No Sampling year was available for the sequences from Greece and for one Yugoslavian strain. B. The tree is the same as the one given in Figure 1B for HIV-1B pol sequences.
(0.27 MB PPT)

Figure S2 Detailed distribution of likelihood mapping of HIV-1A and B pol sequences. The likelihood mappings are the same as the ones reported in Figure 2. The detailed distribution of dots in each region of the map is given as percentage values. Seven main regions can be distinguished: the three corners representing tree-like signal; the three sides representing network-like signal, and the center representing star-like signal. A. Likelihood mapping of 10,000 random quartets of HIV-1A (left) and HIV-1B (right) Albanian sequences. B. Likelihood mapping of 10,000 random quartets of HIV-1A (left) and HIV-1B (right) Albanian+reference sequences downloaded from the Los Alamos HIV databases.
(0.10 MB PPT)

Figure S3 Maximum parsimony gene-flow analysis of HIV-1 subtype A and B in Albania. Rooted trees were estimated by maximum likelihood with the best fitting nucleotide substitution model and a molecular clock taking into account different sampling dates (see Materials and Methods). Edges are drawn not proportional to genetic distances. Each color represents the geographic location of a city as north, south or west of Tirane (the capital, at the center), according to the color legend to the right. The color of a tip branch indicates from which city the actual sequence was sampled. The color of an internal branch is the city origin of the ancestral sequences at the top of that branch, as inferred by the maximum parsimonious reconstruction of ancestral states. A. HIV-1A pol sequences from Albania. B. HIV-1B pol sequences from Albania.
(0.05 MB PPT)
