# Supplementary material for: High-Resolution Molecular Epidemiology and Evolutionary History of HIV-1 Subtypes in Albania
Source: PLoS One. 2008 Jan 2;3(1):e1390. doi: 10.1371/journal.pone.0001390 (PMC2148102; doi:10.1371/journal.pone.0001390)
Supplement: Table S1 — HIV-1 pol gene data sets assembled for the present study. (0.06 MB DOC) [file pone.0001390.s001.doc]

**Table S1. HIV-1 *pol*** gene data sets assembled for the present study.

| Dataset | # sequences | Sampling interval (in years) | Best fitting Markov model^2^ | Shape parameter (α) of Γ-distribution^3^ | Pinv |
| --- | --- | --- | --- | --- | --- |
| HIV-1A Albania | 31 | 1998-2003 | (a b a a b a) + I | - | 0.81 |
| HIV-1A Albania + reference sequences^1^ | 152 | 1990-2005 | (a b a a b a) + Γ | 0.359 | - |
| HIV-1B Albania | 21 | 1995-2002 | (a b c c b c) + Γ + I | 0.445 | 0.62 |
| HIV-1B Albania + reference sequences^1^ | 67 | 1983-2004 | (a b c d b d) + Γ | 0.248 | - |

**1.** Reference sequences of the specific subtype were downloaded from the Los Alamos HIV databases and include strains from Europe and Africa for subtype A, and European strains from subtype B. **2.** Each letter given in parenthesis corresponds to a relative rate, R, of nucleotide substitution for the time-reversible Markov model selected by the hierarchical likelihood ratio test [29] according to the following order: R(A→C), R(A→G), R(A→T), R(C→G), R(C→T), R(G→T). The GTR (most complex time reversible) model, where each rate is different, would be represented by (a b c d e f). Two or more entries in parenthesis with the same letter indicate that the corresponding rates were not significantly different in the selected (simpler) model. For each model relative rates were estimated by maximum likelihood (ML) as described in the text. **3.** Γ-models include with Γ-distributed substitution rates across sites. The α shape parameter of the Γ-distribution was estimated by ML. **4.** I-models include a proportion of invariable sites, Pinv, estimated by ML.
