# Supplementary material for: High-Resolution Molecular Epidemiology and Evolutionary History of HIV-1 Subtypes in Albania
Source: PLoS One. 2008 Jan 2;3(1):e1390. doi: 10.1371/journal.pone.0001390 (PMC2148102; doi:10.1371/journal.pone.0001390)
Supplement: Table S2 — Marginal Likelihoods for different epidemiological models of HIV-1A and B in Albania. (0.04 MB DOC) [file pone.0001390.s002.doc]

Table S2. Marginal Likelihoods* for different epidemiological models of HIV-1A and B in Albania.

| HIV-1 suptype | Constant (strict clock) | Constant (relaxed clock) | Exponential (strict clock) | Exponential (relaxed clock) | BSP (strict clock) | BSP (relaxed clock) |
| --- | --- | --- | --- | --- | --- | --- |
| A | -2788.15±0.23 | -2764.85±0.29 | -2792.31±0.26 | -2760.56±0.22 | -2785.89±0.21 | -2761.67±0.25 |
| B | -2764.85±0.29 | -2754.85±0.29 | -2760.56±0.23 | -2750.56±0.23 | -2761.67±0.25 | -2751.67±0.25 |

* Marginal likelihoods were calculated via importance sampling using the harmonic mean of the sampled likelihoods (with the posterior as the importance distribution).
