# Supplementary figures and images for: High-Resolution Molecular Epidemiology and Evolutionary History of HIV-1 Subtypes in Albania
Source: PLoS One. 2008 Jan 2;3(1):e1390. doi: 10.1371/journal.pone.0001390 (PMC2148102; doi:10.1371/journal.pone.0001390)

## Slide 1
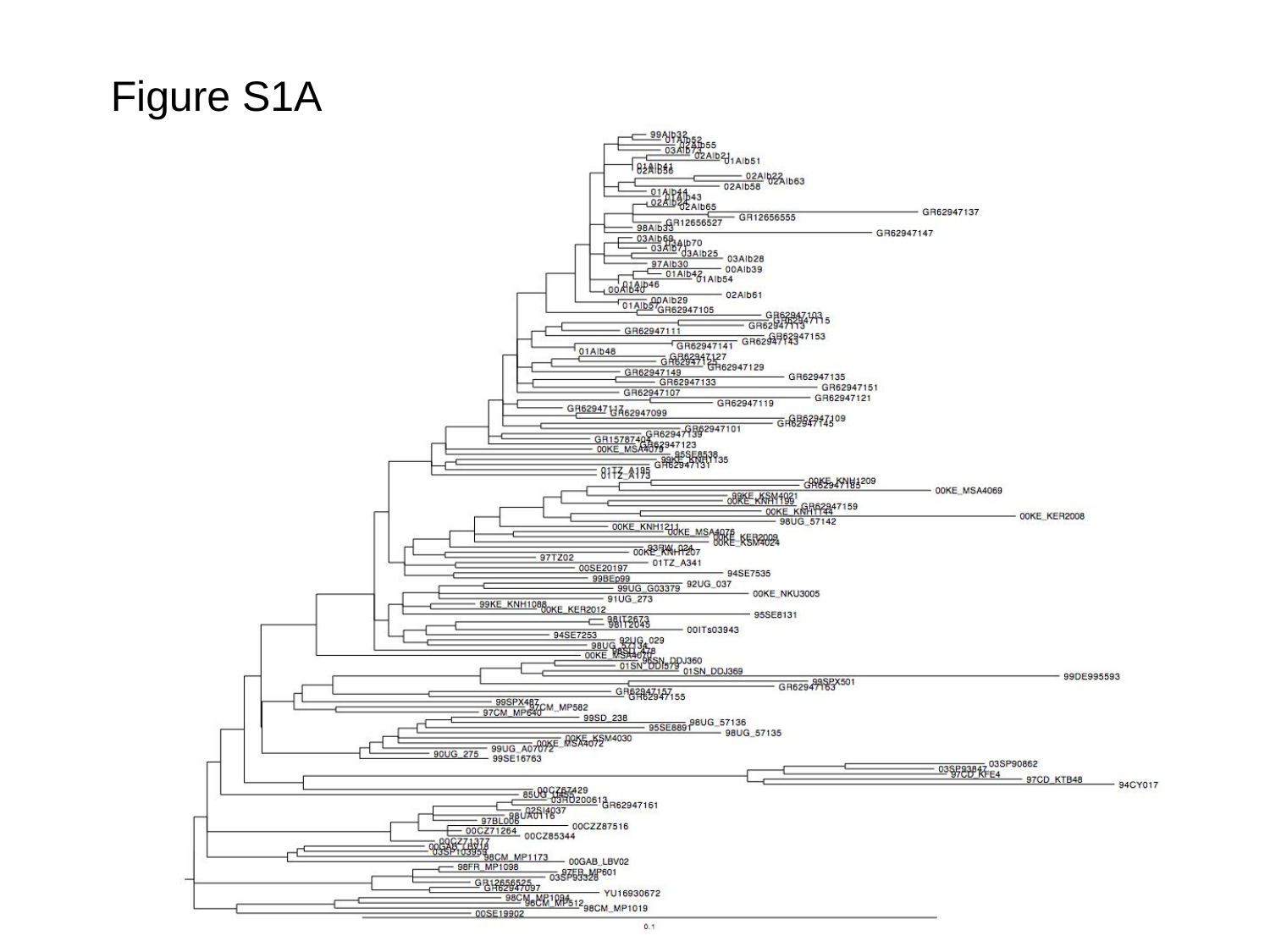

Figure S1A

## Slide 2
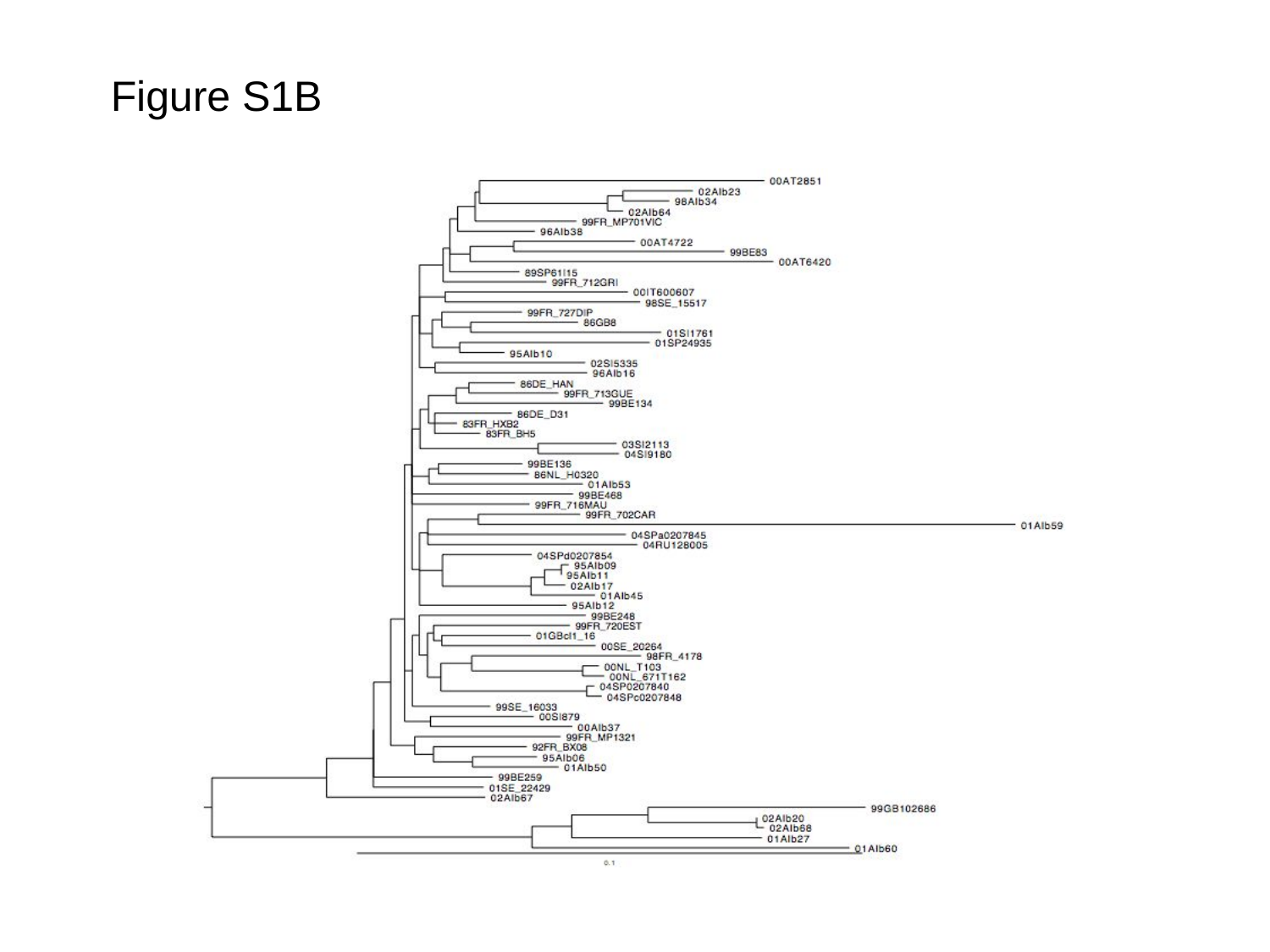

Figure S1B

Supplement: Figure S1 — Maximum likelihood phylogenetic analysis of HIV-1 A and B pol sequences. The maximum likelihood trees display the full names of the HIV-1 sequences used in the analysis. Two digits at the beginning of the name indicate the sampling year, and the next two characters the country of origin according to the guidelines at the Los Alamos HIV databases. Albanian sequences are indicated as “Alb”. A. The tree is the same as the one given in Figure 1A for HIV-1A pol sequences. No Sampling year was available for the sequences from Greece and for one Yugoslavian strain. B. The tree is the same as the one given in Figure 1B for HIV-1B pol sequences. (0.27 MB PPT) [file pone.0001390.s003.ppt]

## Slide 1
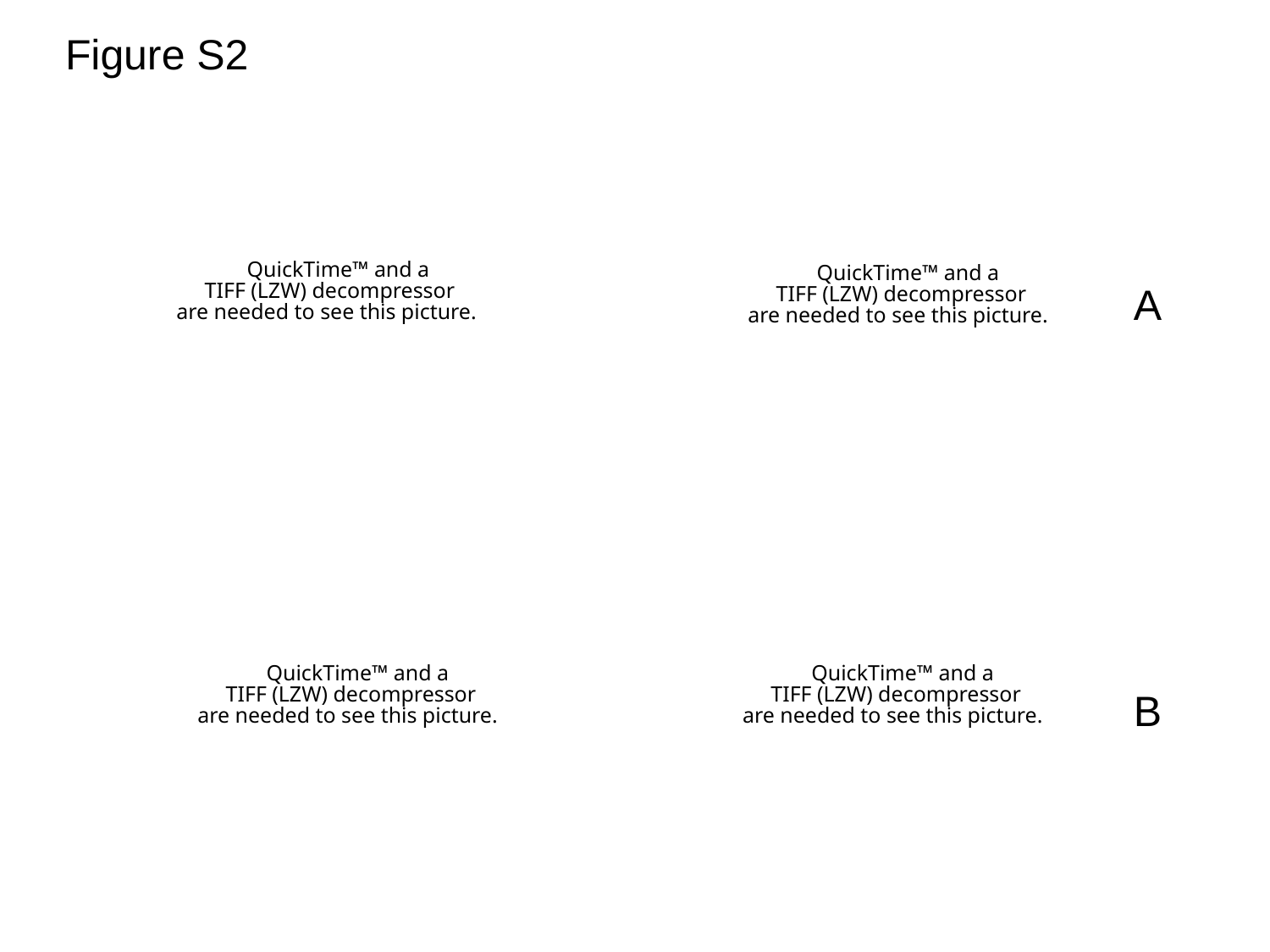

Figure S2
A
B

Supplement: Figure S2 — Detailed distribution of likelihood mapping of HIV-1A and B pol sequences. The likelihood mappings are the same as the ones reported in Figure 2. The detailed distribution of dots in each region of the map is given as percentage values. Seven main regions can be distinguished: the three corners representing tree-like signal; the three sides representing network-like signal, and the center representing star-like signal. A. Likelihood mapping of 10,000 random quartets of HIV-1A (left) and HIV-1B (right) Albanian sequences. B. Likelihood mapping of 10,000 random quartets of HIV-1A (left) and HIV-1B (right) Albanian+reference sequences downloaded from the Los Alamos HIV databases. (0.10 MB PPT) [file pone.0001390.s004.ppt]
