# Supplementary material for: High-Resolution Molecular Epidemiology and Evolutionary History of HIV-1 Subtypes in Albania
Source: PLoS One. 2008 Jan 2;3(1):e1390. doi: 10.1371/journal.pone.0001390 (PMC2148102; doi:10.1371/journal.pone.0001390)
Supplement: Figure S3 — Maximum parsimony gene-flow analysis of HIV-1 subtype A and B in Albania. Rooted trees were estimated by maximum likelihood with the best fitting nucleotide substitution model and a molecular clock taking into account different sampling dates (see Materials and Methods). Edges are drawn not proportional to genetic distances. Each color represents the geographic location of a city as north, south or west of Tirane (the capital, at the center), according to the color legend to the right. The color of a tip branch indicates from which city the actual sequence was sampled. The color of an internal branch is the city origin of the ancestral sequences at the top of that branch, as inferred by the maximum parsimonious reconstruction of ancestral states. A. HIV-1A pol sequences from Albania. B. HIV-1B pol sequences from Albania. (0.05 MB PPT) [file pone.0001390.s005.ppt]

## Slide 1
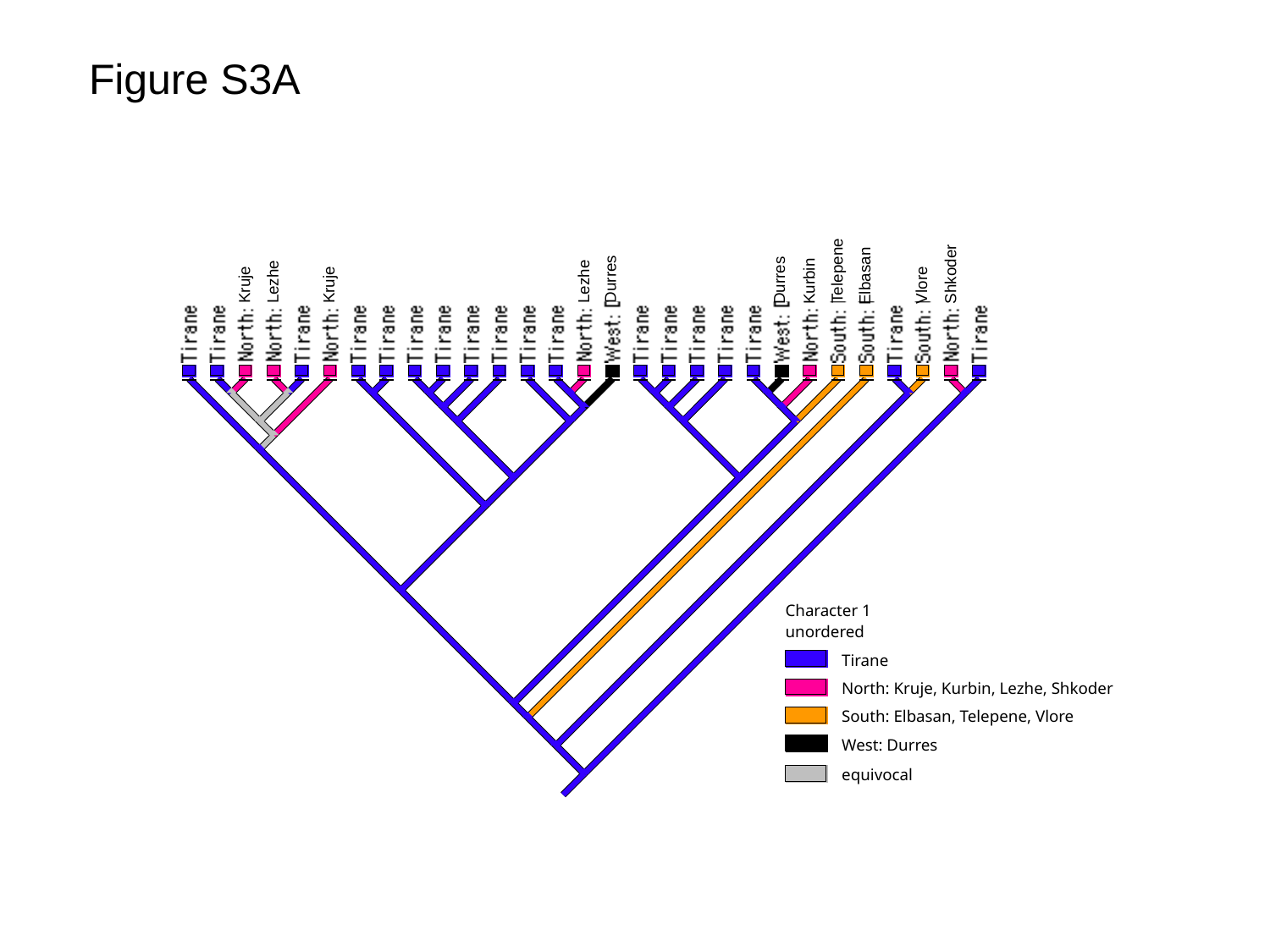

Figure S3A
Telepene
Shkoder
Elbasan
Durres
Durres
Kurbin
Lezhe
Lezhe
Kruje
Kruje
Vlore

## Slide 2
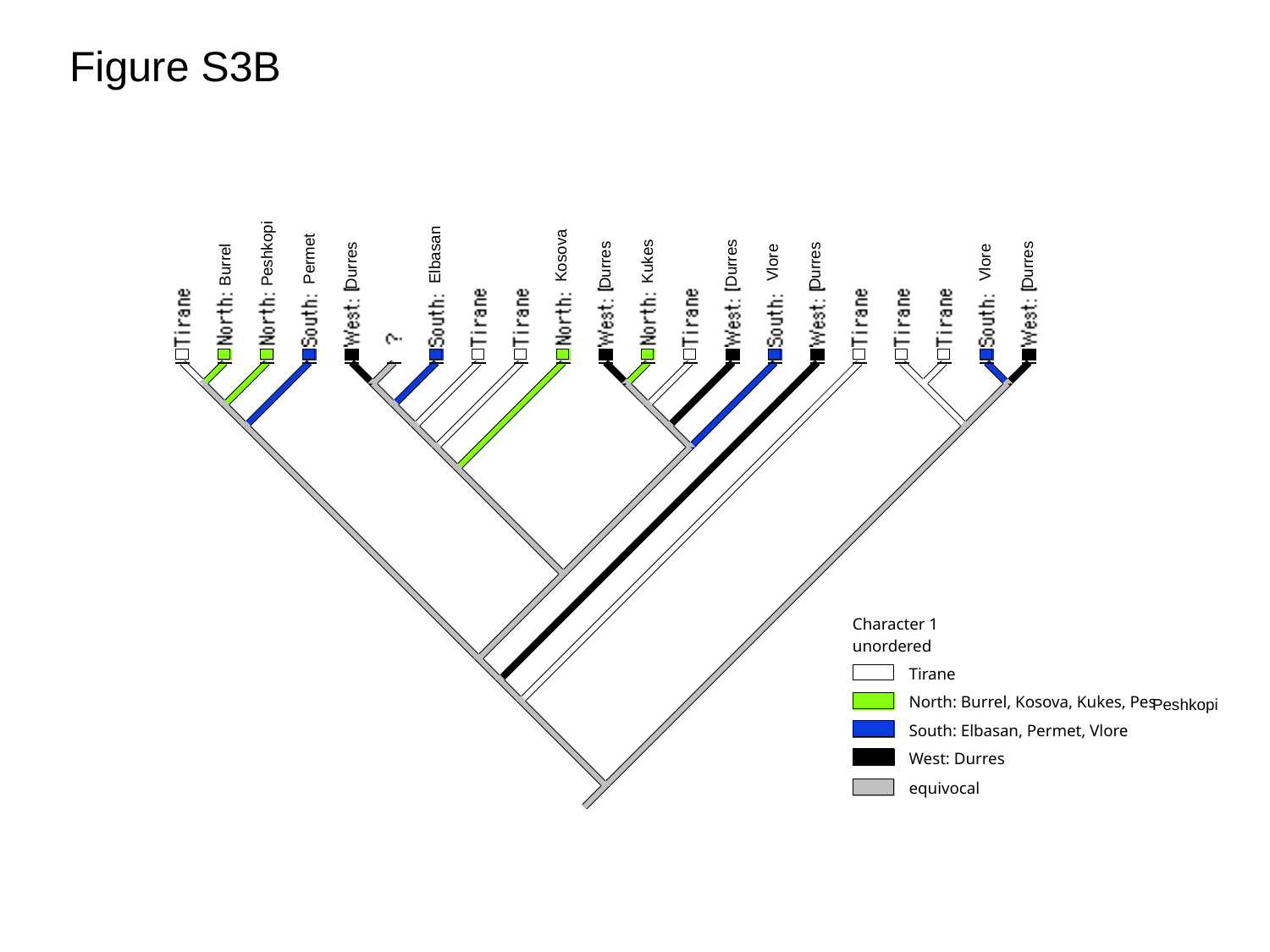

Peshkopi
Figure S3B
Peshkopi
Elbasan
Kosova
Permet
Kukes
Vlore
Vlore
Durres
Burrel
Durres
Durres
Durres
Durres
